# Decrease in Incidence Rate of Hospitalizations Due to AIDS-Defining Conditions but Not to Non-AIDS Conditions in PLWHIV on cART in 2008–2018 in Italy

**DOI:** 10.3390/jcm10153391

**Published:** 2021-07-30

**Authors:** Silvia Nozza, Laura Timelli, Annalisa Saracino, Nicola Gianotti, Claudia Lazzaretti, Alessandro Tavelli, Massimo Puoti, Sergio Lo Caputo, Andrea Antinori, Antonella d’Arminio Monforte, Cristina Mussini, Enrico Girardi, on behalf of ICONA Foundation Study Group

**Affiliations:** 1 Department of Infectious Diseases, San Raffaele Scientific Institute, 20127 Milano, Italy; gianotti.nicola@hsr.it; 2 National Institute for Infectious Diseases L. Spallanzani, IRCCS, 00077 Rome, Italy; laura.timelli@inmi.it (L.T.); andrea.antinori@inmi.it (A.A.); enrico.girardi@inmi.it (E.G.); 3 Infectious Diseases Department, Università degli Studi di Bari, 70126 Bari, Italy; annalisa.saracino@uniba.it; 4 Cilinic of Infectious Diseases, Arcispedale Santa Maria Nuova, 42123 Reggio Emilia, Italy; claudia.lazzaretti@ausl.re.it (C.L.); cristina.mussini@unimore.it (C.M.); 5 ICONA Foundation, 20126 Milano, Italy; alessandro.tavelli@fondazioneicona.org; 6 Department of Infectious Diseases, ASST Grande Ospedale Metropolitano Niguarda, 20126 Milano, Italy; massimo.puoti@ospedaleniguarda.it; 7 Infectious Diseaes Department, University of Foggia, 71122 Foggia, Italy; sergiolocaputo@gmail.com; 8 Department of Health Sciences, Clinic of Infectious Diseases, ASST Santi Paolo e Carlo, University of Milan, 20126 Milano, Italy; antonella.darminio@unimi.it

**Keywords:** hospitalizations, PLWHIV, non-AIDS defining conditions

## Abstract

Background: We aimed to describe the change in the incidence and causes of hospitalization between 2008 and 2018 among persons living with HIV (PLWHIV) who started antiretroviral therapy (ART) from 2008 onwards in Italy. Methods: We included participants in the ICONA (Italian Cohort Naïve Antiretrovirals) cohort who started ART in 2008. All the hospitalizations occurring during the first 30 days from the start of ART were excluded. Hospitalizations were classified as due to: AIDS-defining conditions (ADC), non-ADC infections and non-infections/non-ADC (i.e., cardiovascular, pulmonary, renal-genitourinary, cancers, gastrointestinal-liver, psychiatric and other diseases). Comparisons of rates across time were assessed using Poisson regression. The Poisson multivariable model evaluated risk factors for hospitalizations, including both demographic and clinical characteristics. Results: A total of 9524 PLWHIV were included; 6.8% were drug users, 48.9% men-who-have sex with men (MSM), 39.6% heterosexual contacts; 80.8% were males, 42.3% smokers, 16.6% coinfected with HCV and 6.8% with HBV (HBsAg-positive). During 36,157 person-years of follow-up (PYFU), there were 1058 hospitalizations in 747 (7.8%) persons; they had HIV-RNA >50 copies mL in 34.9% and CD4 < 200/mmc in 27%. Causes of hospitalization were 23% ADC, 22% non-ADC infections, 55% non-infections/non-ADC (11% cancers; 9% gastrointestinal-liver; 6% cardiovascular; 5% renal-genitourinary; 5% psychiatric; 4% pulmonary; 15% other). Over the study period, the incidence rate (IR) decreased significantly (from 5.8 per 100 PYFU in 2008–2011 to 2.21 per 100 PYFU in 2016–2018). Age > 50 years, intravenous drug use (IDU), family history of cardiovascular disease, HIV-RNA > 50, CD4 < 200, were associated with a higher hospitalization risk. Conclusions: In our population of PLWHIV, the rate of hospitalization decreased over time.

## 1. Introduction

Combination antiretroviral therapy (cART) is effective in reducing morbidity and mortality due to HIV infection for people living with HIV (PLWHIV) [1]. Causes and rates of hospitalization depend on considered settings. For example, people with HIV in New York City were more frequently hospitalized than people without HIV; while AIDS-defining illnesses were relatively rare, non-AIDS-defining infection hospitaliza-tions were more common [2]. In Sierra Leone, PLWHIV accounted for a substantial proportion of admissions in hospitals for AIDS-defining conditions, with a high mor-bidity and mortality burden, due to late HIV diagnosis [3].

In Italy in 2018, the proportion of PLWHIV with CD4+ cells count < 200/µL at di-agnosis was 37.8% [4]. The initiation of antiretroviral therapy in HIV-positive adults provides net benefits in the development of AIDS-defining and non-AIDS-defining ill-nesses [5]. Recent data highlight the importance of considering and screening non-AIDS-related conditions [6], which are the principal causes of admission, involv-ing all hospital departments [7].

The observation of causes of hospitalization in PLWHIV is important in our clini-cal practice. The use of antiretroviral therapy decreased AIDS-related events; on the other hand, we observed an increase in median age in PLWHIV.

Our hypothesis was that in the last 10 years, the use of cART with high immuno-virological success was associated with a decrease in hospitalization rates and with a change of causes, with an increase in non-infectious and non-AIDS-related comorbidi-ties, in particular related to age and metabolic parameters.

This study describes incidence rate, causes of hospitalization, factors associated with the risk of hospitalization between 2008 and 2018 of people living with HIV starting ART after 2007.

## 2. Materials and Methods

### 2.1. Study Population

This is a retrospective analysis of prospectively collected data from the ICONA (Italian Cohort Naïve Antiretrovirals) Foundation cohort. ICONA is an observational cohort, set up in 1997, including HIV-1-infected subjects, naïve from ART at the time of enrollment. Demographic, viro-immunological and clinical data and information on antiretroviral regimens are collected and recorded using an electronic database.

All patients signed consent forms to participate in the ICONA Foundation Study in their local participating clinical sites. The research study protocol has been approved by local institutional review boards

We included HIV-infected persons who started first antiretroviral therapy in the period from 1 January 2008 to 31 December 2018, having at least one follow-up visit. For those patients, a hospitalization was considered if it occurred at least 30 days after the enrollment and was defined as admission to any Italian hospital for two or more days. Participants’ follow-up accrued from the date of enrollment (baseline) to their last visit or death or 31th December, 2018 (whichever was earlier). Participants could be included in multiple periods and could contribute more than one hospitalization per period. Socio-demographic factors were collected at baseline, such as sex, age group, nationality, HIV risk factor, family history of cardiovascular disease; HIV-related factors have been collected at baseline and during follow-up (i.e., CD4, HIV viral load, hepatitis C coinfection and time from HIV diagnosis to first ART).

The outcomes evaluated in this study were the rates of hospitalizations for any reason and hospitalizations for specific reasons grouped as follows: AIDS-defining conditions (ADC) (see Supplementary Table S1), infections non-AIDS-defining (including all the infectious parasitic diseases with the exclusion of those classified in ADC group, plus bacterial pneumonia, cellulitis and sepsis, see Supplementary Table S1), and non-infections/non-ADC (i.e., cardiovascular, pulmonary, renal/genitourinary, non-AIDS cancer, liver/gastrointestinal, psychiatric and other diseases, including all other causes not classified in the previously specified categories). We also assessed possible factors associated with hospitalizations.

### 2.2. Statistical Analysis

The baseline for this analysis was the date of the start of the first antiretroviral therapy.

Study period hospitalization rates were calculated as the number of hospitaliza-tions occurring during the period divided by the total of person-years of follow-up (PY). For each participant, the PY at risk were computed from the starting day of the first ART to the last follow-up or death or 31 December, 2018 (whichever was earli-er). Participants could be included in multiple time periods and could contribute more than one hospitalization per time period.

The number of hospitalizations, PY at risk and incidence rate (IR) of hospitaliza-tion (per 100 PY) were calculated overall and for three study periods (every four years and the last period has three years): 2008–2011, 2012–2015 and 2016–2018.

Crude and adjusted incidence rate ratios (IRR) and 95% confidence intervals were calculated using Poisson regression models, and the overdispersion of data was as-sessed with deviance and Pearson goodness-of-fit tests.

Comparisons of the rates across the study period were assessed using a univaria-ble Poisson regression model clustered on person, which adjusts for within-patient correlation. Moreover, the multivariable Poisson regression model was used to assess potential factors for hospitalization. These included both demographic and clinical characteristics, such as sex, age group (i.e., stratified: ≤35, 36–42, 43–51, ≥52 years), na-tionality (Italian, other), HIV risk factor (heterosexual, IDU, MSM, other/unknown), cardiovascular familiarity (yes, no, unknown), CD4 (cell/mm^3^) (≤200, 201–350, 351–500, >500, missing), HIV viral load (copies/mL) (≤50, 51–10,000, >10,000, missing), hepatitis C coinfection (positive, negative, unknown) and time from HIV diagnosis to first ART (months) (0–2, 2–20, >20, based on percentile.

Time-varying characteristics (as CD4 cells, viral load and HCV coinfection) were updated at each time visit.

All factors included in this analysis were also evaluated using a more parsimoni-ous model-building strategy (i.e., inclusion of factors with a log-likelihood ratio test *p*-value < 0.2 at univariate analysis and then backward selection with a log-likelihood ratio test *p*-value < 0.2 at each step). However, we decided to report the model with all covariates, given that the results were similar when using this approach (data not shown).

Moreover, we performed the same analysis using hospitalization or death as the outcome.

Aiming to evaluate an effect modification of each factor in the different study pe-riods, we performed models with interaction terms between each factor and study pe-riods only for any reason of hospitalization.

All the analyses were performed with STATA (version 15).

## 3. Results

### 3.1. Baseline Patients’ Characteristics

We included 9524 naïve participants in the ICONA cohort who started cART between 2008 and 2018: of whom 2166 (22.7%) between 2008 and 2011, 4322 (45.4%) between 2012–2015 and 3036 (31.9%) between 2016 and 2018.

They were followed for 36,157 person-years. Median length of follow-up during this period was 3.3 years (interquartile range (IQR): 1.5–5.7). Men were 7695 (80.8%), 77.9% (*n* = 7414) reported to be Italian. MSM represented 48.9% (*n* = 4571) of the study population, IDU 6.8% (*n* = 647) and heterosexual contact 38.6% (*n* = 3675). Coinfected with hepatitis C were 9.8% (*n* = 937) and 4.5% (*n* = 430) with hepatitis B. Family history of cardiovascular diseases was reported for 14.6% (*n* = 1393) of participants. Median time since the first positive HIV test was 2.3 months (IQR: 0.8–21.2). The median age at enrollment was 39 years (IQR: 32–48), 27% had CD4 count <200 and the vast majority (75.5%) had HIV VL >10,000 (Table 1).

The hospitalized (*n* = 747) compared with non-hospitalized (*n* = 8777) patients had a longer follow-up in years (median 4.7, IQR 2.5–7.0 vs. 3.2, IQR 1.5–5.6), were older (median 43 years, IQR 35–51 vs. 39, IQR 31–47), were more frequently women (25.4% vs. 18.7%), heterosexual and IDU (43.5% vs. 38.2 and 12.4% vs. 6.3%, respectively) and HCV coinfected (16.6% vs. 9.3%); they had higher zenith HIV viral load (median 5 log10 copies/mL, IQR 4.5–5.6 vs. 4.9, IQR 4.3–5.4), lower nadir CD4 (median 186 cells/mm^c^, IQR 54–322 vs. 308, IQR 163–439) and higher proportion of patients with CD4 ≤ 200 cells/mm^c^ (41.6% vs. 25.8%).

### 3.2. Hospitalizations: Rates, Reasons

Of the 9524 people, 747 (7.8%) were hospitalized at least once during 2008–2018, with 1058 separate hospital admissions. Of those 68% had one admission, 18% had two, 6% had three and 8% had four or more admissions. The median length of stay (LOS) was 10 days (IQR: 5–21).

At hospitalization 34.9% had HIV-RNA > 50 copies mL and 27% CD4 < 200/mm^c^.

The highest number of hospitalization was due to non-infection/non-ADC (55%), 23% of hospitalizations were due to ADC and 22% to non-AIDS-defining infections.

**Figure 1:**
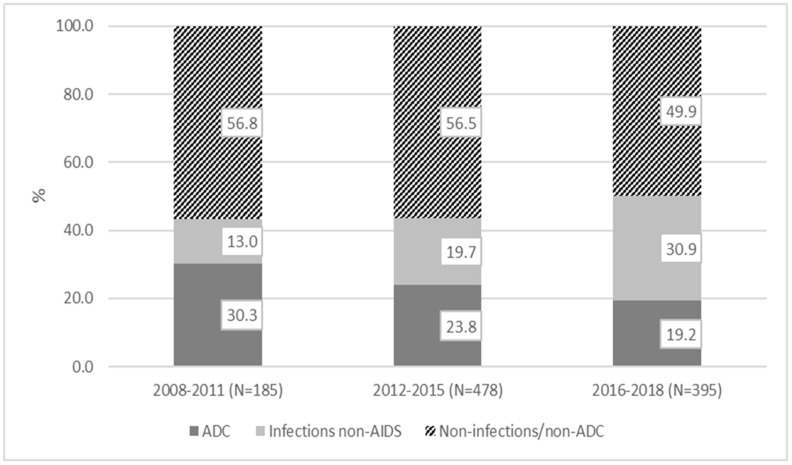
The overall crude rate of hospitalizations during the study period was 2.96 (95% CI: 2.75–3.10) per 100 person-years. It decreased significantly (*p* < 0.001) over the study period from 5.80 (95% CI: 5.02–6.70) in 2008–2011 to 2.21 (95% CI: 2.00–2.44) in 2016–2018. The rates decreased also for ADC, from 1.76 (95% CI: 1.35–2.28) in 2008–2011 to 0.43 (95% CI: 0.34–0.53) in 2016–2018 and for non-infection/non-ADC, from 3.29 (95% CI: 2.72–3.99) in 2008–2011 to 1.10 (95% CI: 0.96–1.27) in 2016–2018, whereas the rate for infections non-AIDS-defining remained stable (Table 2).

Non-infection/non-ADC included oncologic disease (11%), gastrointestinal/liver disease (9%), cardiovascular (6%), renal/genitourinary and psychiatric (5% both), pulmonary (4%) and all other causes not classified in the previously specified categories (15%). Figure 1 shows the distribution of causes of hospitalizations in different time periods.

Observed non-AIDS-defining infections included pneumonia (33%), sexually transmitted infections (26%), sepsis (9%), viral infections (14%), gastrointestinal infections (6%), cutaneous infections (8%) and urinary infections (3%).

The distribution of all the causes making up the definitions of ADC, infections non-AIDS-defining, and non-infections/non-ADC, over time are reported in Supplementary Table S2.

### 3.3. Factors Associated with Hospitalizations

In a multivariable model, older age (≥52 years vs. ≤35, IRR 1.63), being IDU vs. heterosexual (IRR 1.56), family history of cardiovascular disease (IRR 1.30), HIV VL between 51 and 10,000 or >10,000 vs. <50 copies/mL (IRR 1.62 and 1.72, respectively) were associated with a higher risk of hospitalization. On the contrary male sex (RR 0.78), CD4 > 200 (201–350, 351–500 and >500) vs. ≤200 cells/mm^3^ (IRR 0.45, 0.28 and 0.24, respectively), time from HIV diagnosis and starting first ART between 2 and 20 months vs. <2 months (IRR 0.76) and period of hospitalization 2012–2015 and 2016–2018 vs. 2008–2011 (IRR 0.71 and 0.57, respectively) were associated with lower risk of hospitalization (Table 3).

Further when we performed models with interaction terms between each factor and study period, we found significant interactions only for CD4, viral load and mode of infection. However, the effects did not change the direction of the association but the magnitude was increasing as the study period was more recent.

When we performed the same analysis using hospitalization or death as the outcome, we obtained consistent results with those using as the outcome of hospitalizations (see Supplementary Table S3).

The hospitalizations due to ADC were associated with higher levels of viral load, particularly 51–10,000 and >10,000 copies/mL (IRR 1.97 and 2.75, respectively); the non-infection/non-ADC were most associated with other nationality than Italian (IRR 1.45), IDU and MSM (IRR 1.77 and 1.46, respectively) and high viral load, 51–10,000 and >10,000 copies/mL (IRR 1.69 and 1.77, respectively); be over 52 years old, IDU, have a family history of cardiovascular disease and viral load between 51 and 10,000 copies/mL appeared to be associate with a higher risk of hospitalization for infection non-AIDS-defining (IRR 2.02, 1.87, 1.66 and 1.47, respectively). Higher CD4 levels (≥200 cells/mmc) were protective for all reasons (Table 4). 

## 4. Discussion

The current study showed a temporal decrease in the overall hospitalization rate and a decrease in the risk of hospitalization in people living with HIV in care from 2008 to 2018 in the ICONA cohort in care from 2008 to 2018 in the ICONA cohort. After the introduction of cART, the hospitalizations rate declined in PLWHIV, and this decline (−61.8%) was greater than the decline in age-standardized hospitalization rates observed for the general population in the same time period (−26.4%), according to the official register of Italian Health Departement. Non-AIDS-related hospitalizations were more frequent than those related to AIDS [2,6,7,8,9].

A recent editorial underlines the importance of reaching zero hospitalizations to reach zero deaths attributable to HIV [10] and considers every hospitalization as a failure of public health.

When considering different reasons for hospitalization, major declines were detected for AIDS-defining events. This is comparable to other cohorts, and it is due to ART efficacy.

Hospitalizations due to non-infection/non-ADC were stable over time. Published data of the ICONA cohort showed an increase in the prevalence of some non-communicable diseases from 2004 to 2014; in particular, the prevalence of dyslipidemia, hypertension and cardiovascular disease increased [11]. In our study, cardiovascular disease caused 6% of hospitalizations. Oncologic disease caused 11% of hospitalizations; the malignancies in HIV Italian cohorts were observed in 8.5% of the CISAI cohort [12] and in 3.8% of the ICONA cohort [13].

The percentage of hospitalizations due to non-AIDS-defining infections increased during the observation time. The most common condition associated was pneumonia; this observation is consistent with another cohort [14] data; in our cohort, the crude incidence of pneumonia was 5.66 per 1000 person-years from 1996 to 2011 [15]. The second cause of hospitalization was a sexually transmitted infection; in Italy, there was an outbreak of sexually acquired hepatitis A [16], and there is a treatment failure to syphilis in about 10% of PLWHIV [17]; these data could explain the high rate of hospitalizations due to STDs.

The higher risk of hospitalization was associated with older age, use of injecting drugs and detectable viral load. The overall prevalence of multimorbidity in the geriatric Italian GEPPO cohort amounted to 64%, and it is related to longer duration of HIV infection rather than older age per se [18]; the prevalence of comorbidities in PLWHIV is higher than in HIV-negative people, probably due to the inflammation [19]. Early determination of frailty could lead to target interventions to decrease adverse outcomes that cause hospitalization.

The use of injecting drugs could be related to sepsis; the likelihood of bacteremia was found to have increased in a consortium of sites in the USA in the HAART era [20]. In our study, hospitalizations were found more frequently in the group of people with injecting drug use (former or current), not because of active injecting drug use.

Detectable viral load is associated with a higher risk of hospitalization and, in particular, to non-AIDS-defining events due to inflammation-related events [21].

Focus on social factors, low educational grades and unemployed are associated with a higher risk of hospitalization. Several studies have identified limited literacy as a risk factor for poor HIV medication adherence, despite evidence that questions this association [22,23]. These findings are in line with this issue.

This study has some limitations: first, ICONA is not representative of all Italian centers, and some of them are the reference for many clinical and surgical specialties; this could overestimate the hospitalization rate. Another limitation is the classification of hospitalization, with some diseases that could be misunderstood (for example, acute hepatitis infections could be classified as gastrointestinal diseases and sexually transmitted diseases). Moreover, it is of note that we estimated some IRR with very large 95% confidence intervals. This reflects the lack of statistical power for some of the potential determinants.

Our study is useful to understand the changing of hospitalization causes and rates in the era of antiretroviral therapy; we analyzed data of patients treated in the last 10 years considering the high rate of immunovirological success of recent cART, and in this setting, we conclude that prevention strategies against non-communicable diseases and non-AIDS-defining infections are crucial to decrease hospitalization rate. These strategies could include stop smoking, starting a low-calorie diet and use of specific drugs (e.g., statins, hypoglycemic).

## Figures and Tables

**Table 1:** Characteristics of all enrolled patients (between 2008 and 2018)—ICONA cohort.

	All Enrolled		Non-Hospitalized		Hospitalized		
	N = 9524		N = 8777		N = 747		*p*-Value **
Follow-up (years), median (IQR)	3.3	1.6–5.7	3.2	1.5–5.6	4.7	2.5–7.0	<0.01
Sex, n (%)							
Female	1829	1.2	1639	18.7	190	25.4	<0.01
Male	7695	80.8	7138	81.3	557	74.6	
Age at first ART (years), median (IQR)	39	32–48	39	31–47	43	35–51	<0.01
Ethnicity, n (%)							
Asian	116	1.2	109	1.2	7	0.9	0.637
Black	815	8.6	742	8.5	73	9.8	
Caucasian	7901	83	7289	83	612	81.9	
Hispanic/Latino	509	5.3	466	5.3	43	5.8	
Other/unknwon	183	2	171	1.9	12	1.6	
Nationality, n (%)							
Italian	7414	77.9	6838	77.9	576	77.1	0.614
Other	2110	22.2	1939	22.1	171	22.9	
HIV risk factor, n (%)							
Heterosexual	3675	38.6	3350	38.2	326	43.5	<0.01
IDU	647	6.8	554	6.3	93	12.4	
MSM	4572	48	4299	49	273	36.5	
Other/Unknown	630	6.6	574	6.5	56	7.5	
Education, n (%)							
Primary	444	4.7	386	4.4	58	7.8	<0.01
Secondary	1544	16.2	1379	15.7	165	22.1	
High	2845	29.9	2661	30.3	184	24.6	
Degree	1196	12.5	1117	12.7	79	10.6	
Unknown	3495	36.7	3234	36.9	261	34.9	
Occupation, n (%)							
Housewife	183	1.9	152	1.7	31	4.1	<0.01
Unemployed	1162	12.2	1.044	11.9	118	15.8	
Disabled	21	0.2	19	0.2	2	0.3	
Self-employed	1261	13.2	1182	13.5	79	10.6	
Employee	3764	39.5	3482	39.7	282	37.7	
Occasional worker	263	2.8	239	2.7	24	3.2	
Retired	261	2.7	213	2.4	48	6.4	
Student	320	3.4	311	3.5	9	1.2	
Other/Unknown	2289	24	2.135	24.3	154	20.6	
Smoke use, n (%)							
No smoker	4830	50.7	4475	51	355	47.5	0.177
Smoker	3800	39.9	3480	39.7	320	42.8	
Unknown	894	9.4	822	9.4	72	9.6	
Alcohol use, n (%)							
Abstainer	3999	42	3647	41.6	352	47.1	<0.01
Abuse	14	0.2	14	0.2	0	0	
Drinker	3509	36.8	3271	37.3	238	31.9	
Unknown	2002	21	1845	21	158	21	
Family history of cardiovascular disease, n (%)							
No	5178	54.4	4785	54.5	393	52.6	0.405
Yes	1393	14.6	1287	14.7	106	14.2	
Unknown	2953	31	2705	30.8	248	33.2	
Hepatitis C *, n (%)							
Negative	7925	83.2	7341	83.6	584	78.2	<0.01
Positive	937	9.8	813	9.3	124	16.6	
Unknown	662	7	623	7.1	39	5.2	
Hepatitis B *, n (%)							
Negative	8053	8.6	7.414	84.5	639	85.5	<0.01
Positive	430	4.5	379	4.3	51	6.8	
Unknown	1041	10.9	984	11.2	57	7.6	
Zenith HIV viral load (log10 copies/mL), median (IQR)	4.9	4.3–5.4	4.9	4.3–5.4	5	4.5–5.6	<0.01
Nadir CD4, median (IQR)	300	150–433	308	163–439	186	54–322	<0.01
HIV viral load copies/mL (classes) *, n (%)							
≤50	201	2.1	186	2.1	15	2	0.671
51–10,000	2099	22	1.947	22.2	152	20.4	
>10,000	7187	75.5	6.609	75.3	578	77.4	
Missing	37	0.4	35	0.4	2	0.3	
HIV viral load (copies/mL) *, median (IQR)	48,959	10,756–186,200	48,168	10,584–181,170	61,830	12,675–264,700	
CD4 cell count (classes) *, n (%)							
≤200	2571	27	2260	25.8	311	41.6	<0.01
201–350	1861	19.5	1727	19.7	134	17.9	
351–500	2032	21.3	1918	2.9	114	15.3	
>500	3032	3.8	2846	3.4	186	24.9	
Missing	28	0.3	26	0.3	2	0.3	
CD4 (cell count) *, median (IQR)	371	183–560	379	193–566	268	77–500	<0.01
Time from HIV diagnosis to first ART (months), median (IQR)	2.3	0.8–21.2	2.3	0.8–20.4	2.1	0.7–34.3	0.702

Note: * at enrollment in ICONA cohort. ** Chi-square/Fisher exact test or Mann—Whitney test, as appropriate.

**Table 2:** Hospitalization rates, overall and for grouping, per period.

	Time at Risk (Years)	Subjects	Number of Hospitalizations	IR * 100	95%CI		*p*-Value
					L	U	
All hospitalizations							
2008–2011	3191	2187	185	5.80	5.02	6.70	ref
2012–2015	15,118	6400	478	3.16	2.89	3.46	<0.01
2016–2018	17,858	8687	395	2.21	2.00	2.44	<0.01
AIDS-defining conditions							
2008–2011	3191	2187	56	1.76	1.35	2.28	ref
2012–2015	15,118	6400	114	0.75	0.63	0.91	<0.01
2016–2018	17,858	8687	76	0.43	0.34	0.53	<0.01
Infections non-ADC							
2008–2011	3191	2187	24	0.75	0.50	1.12	ref
2012–2015	15,118	6400	94	0.62	0.51	0.76	0.428
2016–2018	17,858	8687	122	0.68	0.57	0.82	0.68
Non-infections/non-ADC							
2008–2011	3191	2187	105	3.29	2.72	3.98	
2012–2015	15,118	6400	270	1.79	1.59	2.01	<0.01
2016–2018	17,858	8687	197	1.10	0.96	1.27	<0.01

Note: *p*-values were obtained from Log-rank test.

**Table 3:** Univariate and Multivariable analysis of factors associated with hospitalization—Poisson regression model.

		Univariate				Multivariable			
	Factors	IRR	*p*-Value	(5% Conf.	Interval)	IRR	*p*-Value	(95% Conf	Interval)
Period	2008–2011 (ref.)								
	2012–2015	0.55	0.000	0.42	0.71	0.71	0.019	0.54	0.95
	2016–2018	0.38	0.000	0.29	0.5	0.57	0.000	0.42	0.76
Sex	Male vs. Female	0.69	0.001	0.55	0.85	0.78	0.052	0.61	1.00
Class age (years)	≤35 (ref.)								
	36–42	1.1	0.519	0.83	1.45	1.07	0.642	0.81	1.40
	43–51	1.27	0.060	0.99	1.63	1.19	0.161	0.93	1.52
	≥52	1.76	0.000	1.37	2.26	1.63	0.000	1.28	2.08
Natonality	Other vs. Italian	1.19	0.116	0.96	1.48	1.13	0.280	0.91	1.40
HIV risk factor	Heterosexual (ref.)								
	IDU	1.99	0.000	1.46	2.71	1.56	0.010	1.11	2.19
	MSM	0.74	0.003	0.61	0.9	1.15	0.235	0.91	1.46
	Other/Unknown	1.01	0.973	0.72	1.41	0.99	0.954	0.71	1.38
Family history of cardiovascular disease	No (ref.)								
	Yes	1.33	0.005	1.09	1.63	1.30	0.010	1.07	1.60
	Unknown	0.82	0.110	0.64	1.05	0.84	0.168	0.66	1.08
CD4 (cells/mm^3^)	≤200 (ref.)								
	201–350	0.4	0.000	0.32	0.5	0.45	0.000	0.35	0.57
	351–500	0.23	0.000	0.18	0.28	0.29	0.000	0.23	0.37
	>500	0.16	0.000	0.13	0.19	0.24	0.000	0.19	0.30
	Missing	0.47	0.023	0.24	0.9	0.48	0.313	0.12	2.00
HIV viral load (copies/mL)	≤50 (ref.)								
	51–10,000	2.39	0.000	1.99	2.88	1.62	0.000	1.32	1.97
	>10,000	3.43	0.000	2.77	4.27	1.72	0.000	1.35	2.20
	Missing	2.45	0.003	1.36	4.42	1.59	0.490	0.43	5.93
Hepatitis C coinfection	Negative (ref.)								
	Positive	1.55	0.037	1.03	2.33	1.03	0.900	0.66	1.59
	Unknown	0.7	0.022	0.51	0.95	0.73	0.036	0.55	0.98
Time from HIV diagnosis to first ART (months)	0–2 (ref.)								
	2–20	0.52	0.000	0.42	0.65	0.76	0.019	0.60	0.96
	>20	0.92	0.436	0.74	1.14	1.15	0.219	0.92	1.44

**Table 4:** Multivariable analysis of factors associated with grouping hospitalizations—Poisson regression model.

Factors		ADC				Infection NON-AIDS-Defining				Non-Infection/NON-ADC			
		IRR	P > z	95% CI		IRR	P > z	95% CI		IRR	P > z	95% CI	
Period	2008–2011 (ref.)												
	2012–2015	0.69	0.150	0.42	1.14	1.10	0.696	0.67	1.80	0.64	0.007	0.46	0.89
	2016–2018	0.49	0.011	0.28	0.85	1.44	0.162	0.86	2.41	0.42	0.000	0.30	0.59
Sex	Male vs. Female	1.25	0.322	0.81	1.93	0.60	0.009	0.41	0.88	0.71	0.036	0.52	0.98
Class age (years)	≤35 (ref.)												
	36–42	1.38	0.268	0.78	2.44	1.08	0.707	0.72	1.61	0.93	0.679	0.65	1.32
	43–51	1.35	0.171	0.88	2.09	1.08	0.695	0.73	1.60	1.18	0.316	0.85	1.65
	≥52	1.39	0.153	0.89	2.17	1.06	0.777	0.70	1.60	2.02	0.000	1.45	2.80
Nationality	Other vs Italian	1.20	0.362	0.81	1.77	1.45	0.029	1.04	2.03	0.95	0.748	0.71	1.28
HIV risk factor	Heterosexual (ref.)												
	IDU	0.72	0.316	0.37	1.38	1.77	0.027	1.07	2.94	1.87	0.005	1.21	2.88
	MSM	1.18	0.465	0.76	1.84	1.46	0.047	1.00	2.12	1.05	0.754	0.78	1.41
	Other/Unknown	1.00	0.997	0.55	1.81	0.66	0.245	0.33	1.32	1.11	0.602	0.75	1.63
Family history of cardiovascular disease	No (ref.)												
	Yes	0.94	0.764	0.64	1.39	1.02	0.909	0.74	1.39	1.66	0.000	1.30	2.13
	Unknown	0.45	0.018	0.23	0.87	1.04	0.863	0.70	1.54	0.96	0.802	0.70	1.32
CD4 (cells/mm^3^)	≤200 (ref.)												
	201–350	0.23	0.000	0.14	0.39	0.77	0.269	0.49	1.22	0.57	0.000	0.42	0.78
	351–500	0.10	0.000	0.06	0.18	0.39	0.000	0.23	0.66	0.45	0.000	0.32	0.63
	>500	0.06	0.000	0.03	0.10	0.35	0.000	0.22	0.58	0.37	0.000	0.27	0.52
	Missing	0.87	0.899	0.10	7.46	0.32	0.145	0.07	1.49	0.32	0.050	0.10	1.00
HIV viral load (copies/mL)	≥50 (ref.)												
	51–10,000	1.97	0.001	1.33	2.94	1.69	0.008	1.14	2.49	1.47	0.003	1.14	1.89
	>10,000	2.75	0.000	1.80	4.20	1.77	0.018	1.10	2.83	1.20	0.304	0.85	1.70
	Missing	0.93	0.945	0.11	7.83	2.97	0.145	0.69	12.82	2.14	0.137	0.78	5.86
Hepatitis C coinfection	Negative (ref.)												
	Positive	1.18	0.742	0.44	3.12	1.38	0.300	0.75	2.52	0.93	0.798	0.53	1.62
	Unknown	1.05	0.898	0.52	2.10	0.90	0.671	0.55	1.48	0.62	0.008	0.44	0.88
Time from HIV diagnosis to first ART (months)	0–2 (ref.)												
	2–20	0.41	0.002	0.23	0.72	0.76	0.185	0.51	1.14	0.92	0.560	0.69	1.22
	>20	1.16	0.537	0.73	1.85	1.35	0.084	0.96	1.90	1.11	0.442	0.85	1.47

## Data Availability

Not applicable.

## Supplementary Materials

The following are available online at https://www.mdpi.com/article/10.3390/jcm10153391/s1, Table S1: ICD9 codes used to group hospitalizations, Table S2: Distribution of all the causes making up the definitions of ADC, infections non-AIDS defining, and non-infections/non-ADC over time, Table S3: Multivariable analysis of factors associated with hospitalization or death-Poisson regression model.
