# Supplementary material for: Decrease in Incidence Rate of Hospitalizations Due to AIDS-Defining Conditions but Not to Non-AIDS Conditions in PLWHIV on cART in 2008–2018 in Italy
Source: J Clin Med. 2021 Jul 30;10(15):3391. doi: 10.3390/jcm10153391 (PMC8347382; doi:10.3390/jcm10153391)
Supplement: Supplementary file 1 [file jcm-10-03391-s001.zip › jcm-1283887-supplementary.pdf]

Supplementary Table S1. ICD9 codes used to group hospitalizations.

	ICD-9 codes
**AIDS defining condition**	
Candidiasis of lungs	112.4
Candidiasis, esophageal	112.84
Cervical cancer, invasive	180.0-180.9
Coccidioidomycosis, disseminated or extrapulmonary	114.1-114.4
Cryptococcosis, extrapulmonary	117.5, 321.0
Cryptosporidiosis, chronic intestinal	007.4
Cytomegalovirus disease (other than liver, spleen, or nodes)	078.5
Encephalopathy, HIV-related	348.3, 348.30, 348.39
Herpes simplex: chronic ulcer(s); or bronchitis, pneumonitis, or esophagitis	054.1-0.54.19, 054.71, 054.79
Histoplasmosis, disseminated or extrapulmonary	115 escluding 115.05, 115.15, 1153.95
Isosporiasis, chronic intestinal	007.2
Kaposi's sarcoma	176.0-176.9
Lymphoma, Burkitt's	200.20-200.28
Lymphoma, immunoblastic	200.00-200.08
Lymphoma, primary, of brain	200.50-200.58
Mycobacterium avium complex or M. kansasii, disseminated or extrapulmonary	031.2
Mycobacterium tuberculosis, any site (pulmonary or extrapulmonary)	010.00-018.96
Mycobacterium, other species or unidentified species, disseminated or extrapulmonary	031.8, 031.9
Pneumocystis carinii pneumonia	136.3
Pneumonia, recurrent	481.0-483.1, 484.3-484.5, 484.8-486
Progressive multifocal leukoencephalopathy	046.3
Salmonella septicemia, recurrent	003.1
Toxoplasmosis of brain	130.0, 130.7, 130.9
Wasting syndrome due to HIV	783.21, 783.22, 783.7, 260-263.9
**Infection Non-AIDS defining**	
Infectious parasitic diseases	001-139 excluding those classified in AIDS defining illnesses
Bacterial pneumonia	481.0-483.1, 484.3-484.5, 484.8-486
Cellulitis	682.0-682.9
Sepsis	995.91-995.92

Supplementary Table S2. Distribution of all the causes making up the definitions of ADC, infections non-AIDS defining, and non-infections/non-ADC over time

	2008-2011		2012-2015		2016-2018	
	n	%	n	%	n	%
**AIDS defining condition**	56		114		76	
Candidiasis, esophageal	2	3.57	3	2.63	2	2.63
Cervical cancer, invasive	1	1.79	1	0.88	0	0
Cryptococcosis, extrapulmonary	2	3.57	7	6.14	1	1.32
Cryptosporidiosis, chronic intestinal	1	1.79	1	0.88	0	0
Cytomegalovirus disease (other than liver, spleen, or nodes)	1	1.79	3	2.63	8	10.53
Encephalopathy, HIV-related	2	3.57	2	1.75	3	3.95
Herpes simplex: chronic ulcer(s); or bronchitis, pneumonitis, or esophagitis	2	3.57	0	0	1	1.32
Kaposi's sarcoma	8	14.29	15	13.16	6	7.89
Lymphoma, Burkitt's	14	25	9	7.89	8	10.53
Lymphoma, immunoblastic	3	5.36	17	14.91	4	5.26
Lymphoma, primary, of brain	1	1.79	3	2.63	1	1.32
Mycobacterium avium complex or M. kansasii, disseminated or extrapulmonary	3	5.36	3	2.63	4	5.26
Mycobacterium tuberculosis, any site (pulmonary or extrapulmonary)	5	8.93	14	12.28	16	21.05
Mycobacterium, other species or unidentified species, disseminated or extrapulmonary	4	7.14	4	3.51	3	3.95
Pneumocystis carinii pneumonia	0	0	2	1.75	6	7.89
Progressive multifocal leukoencephalopathy	1	1.79	14	12.28	6	7.89
Toxoplasmosis of brain	1	1.79	8	7.02	5	6.58
Wasting syndrome due to HIV	0	0	6	5.26	1	1.32
Other	5	8.93	2	1.75	1	1.32
**Infection Non-AIDS defining**	24		94		122	
Other infections	0	0	1	1.06	4	3.28
Bacterial pneumonia	10	41.67	30	31.91	38	31.15
STI	3	12.5	19	20.21	40	32.79
Sepsis	6	25	7	7.45	9	7.38
Viral	2	8.33	16	17.02	15	12.3
Gastrointestinal	1	4.17	10	10.64	4	3.28
Cuteneus	1	4.17	10	10.64	8	6.56
Urinary infection	1	4.17	1	1.06	4	3.28
**Non-infection/non-ADC**	105		270		197	
gastrointestinal/liver disease	11	10.48	48	17.78	34	17.26
cardiovascular	7	6.67	34	12.59	25	12.69
oncologic disease	30	28.57	55	20.37	32	16.24

renal/genitourinary	9	8.57	20	7.41	20	10.15
pulmonary	9	8.57	17	6.3	15	7.61
psychiatric	18	17.14	19	7.04	11	5.58
other causes	21	20	77	28.52	60	30.46

Note: percentages are calculated with respect to the total hospitalizations for the period relating to the 3 reference groups AIDS defining condition, Infection Non-AIDS defining and Non-infection/non-ADC)

Supplementary Table S3. Multivariable analysis of factors associated with hospitalization or death - Poisson regression model

	Factors	IRR	P-value	[95% Conf.	Interval]
Period	2008-2011 (ref.)				
	2012-2015	0.81	0.108	0.62	1.05
	2016-2018	0.63	0.001	0.48	0.83
Gender	Male vs Female	0.80	0.044	0.64	0.99
Class age (years)	<=35 (ref.)				
	36-42	1.11	0.434	0.85	1.45
	43-51	1.34	0.012	1.07	1.68
	>=52	1.96	0.000	1.56	2.45
Natonality	Other vs Italian	1.12	0.278	0.91	1.36
HIV risk factor	Heterosexual (ref.)				
	IDU	1.63	0.001	1.21	2.19
	MSM	1.12	0.314	0.90	1.39
	Other/Unknown	1.07	0.623	0.81	1.42
Family history of cardiovascular disease	no (ref.)				
	yes	1.29	0.008	1.07	1.55
	Unknown	0.87	0.220	0.69	1.09
CD4 (cells/mm^3)	<=200 (ref.)				
	201-350	0.41	0.000	0.33	0.51
	351-500	0.25	0.000	0.20	0.32
	>500	0.21	0.000	0.17	0.26
	Missing	0.47	0.258	0.12	1.75
HIV viral load (copies/ml)	<=50 (ref.)				
	51-10000	1.65	0.000	1.37	1.98
	>10000	2.02	0.000	1.63	2.52
	Missing	1.38	0.610	0.40	4.79
Hepatitis C coinfection	Negative (ref.)				
	Positive	1.10	0.647	0.74	1.62
	Unknown	0.79	0.086	0.61	1.03
Time from HIV diagnosis to first ART (months)	0-2 (ref.)				
	2-20	0.80	0.039	0.64	0.99
	>20	1.14	0.208	0.93	1.41
